# Reconstruction of Complex Soft Tissue Defects of the Heel With Versatile Double Skin Paddle Anterolateral Thigh Perforator Flaps: An Innovative Way to Restore Heel Shape

**DOI:** 10.3389/fsurg.2022.836505

**Published:** 2022-02-14

**Authors:** Jiqiang He, Gunel Guliyeva, Panfeng Wu, Fang Yu, Liming Qing, Juyu Tang

**Affiliations:** ^1^Xiangya Hospital, Central South University, Changsha, China; ^2^The Johns Hopkins Hospital, Johns Hopkins Medicine, Baltimore, MD, United States

**Keywords:** anterolateral thigh perforator flap, double skin paddle, complex soft-tissue defects, heel, reconstructive surgical procedures

## Abstract

**Background:**

Complex heel defects constitute a significant challenge for plastic surgeons.

**Objectives:**

In this study, versatilities of free double skin paddle ALT flaps in the reconstruction of complex soft tissue defects of heels were explored.

**Methods:**

From January 2010 to December 2019, 16 patients (13 male and 3 females) aged 16–74 years underwent double skin paddle ALT flap reconstruction in our department. All the patients had large defects located at the heel, and 5 had a dead space. Underlying structures such as vessels, bones, and tendons were exposed in all cases.

**Results:**

Flap survival rate was 100% after the reconstruction. Eleven double skin paddle ALT flaps and 5 vastus lateralis muscle-chimeric double skin paddle ALT flaps were used. The size of the skin flap ranged from 9.5 × 4.5 cm^2^ to 22 × 10 cm^2^, and the size of a muscle segment ranged from 6 × 3 × 1 cm^3^ to 10 × 3 × 2 cm^3^. The mean follow-up was 22.6 months (range: 10–81 months). The wounds healed well, providing reliable soft tissue coverage and good heel contour. All the patients ambulated independently during the follow-up period. Most of them regained protective sensation. The average two-point discrimination was 32.7 mm (range: 27–37 mm).

**Conclusion:**

Double skin paddle ALT flaps are a feasible option for the reconstruction of complex heel defects, with good functional and aesthetic results. Nonetheless, further studies comparing double skin paddle ALT flaps to other flap techniques are needed.

## Introduction

Reconstruction of large heel defects with or without significant dead space is challenging and often requires complex surgical techniques and advanced microsurgical training (1). Soles bear body weight, and the skin of soles is thick, adherent, and glabrous. When these tissues are damaged, it is hard to find similar tissues to repair the wound (2–4). Besides, heels have a complex shape. Therefore, aesthetic appearance after reconstruction has to be considered in addition to shoe-wearing and walking.

Several flaps have been reported to repair heel defects, such as V-Y advancement flaps, cross-leg flaps, and island pedicle flaps (5–10). However, in extensive defects, these flaps do not give satisfactory results. In those instances, free flaps, such as the anterolateral thigh (ALT) flap and radial forearm flap, are considered (11, 12). Nonetheless, the use of these flaps in the heel area is limited by challenges associated with restoration of heel shape.

To overcome these shortcomings, we employed double skin paddle ALT flaps to reconstruct complex soft-tissue defects of heels. Versatilities of the free double-paddle ALT flaps in a variety of complex heel defects were also explored. To our knowledge, the use of double-skin paddle ALT flaps for the reconstruction of complex heel defects and restoring heel shape has not been widely reported.

## Patients and Methods

### Patients

All surgical procedures were performed in our hand and microsurgery department. This study followed the ethical committee guidelines of our institution, and the protocol was developed in accordance with the ethical standards of the Helsinki Declaration of 1975 and all subsequent revisions. Written informed consent was obtained from all the patients.

From January 2010 to December 2019, 16 patients (13 males and 3 females) underwent heel reconstruction with double skin paddle ALT flaps. All defects were large with exposure of underlying vital structures, such as vessels, bones, and tendons. Wounds with dead space in five of the patients required filling. Patient characteristics are summarized in Table 1.

**Table 1:** Patients' characteristics.

**Case**	**Age (y)/Sex**	**Cause of injure**	**Location of the wound**	**Flap type**	**Dimensions of flaps (cm)**	**Recipient's vessels**	**Donor site**	**Complications**
1	27/M	Snake bite	Right heel	Double skin paddle	Flap 1: 14 × 6.5; Flap 2: 15 × 7	PTA	DC	None
2	25/M	Traffic accident	Right heel	Double skin paddle	Flap 1: 22 × 10; Flap 2: 19 × 9	PTA	DC	None
3	41/M	Traffic accident	Right heel	Double skin paddle	Flap 1: 12 × 8.5; Flap 2: 14 × 7	PTA	DC	None
4	45/M	Traffic accident	Left heel	Flow-through double skin paddle	Flap 1: 20 × 7; Flap 2: 10 × 6	Flow through bridge PTA	DC	None
5	74/F	Traffic accident	Left heel	Vastus lateralis muscle-chimeric double skin paddle	Flap 1: 16 × 6; Flap 2: 9 × 6; Muscle flap: 8 × 3 × 2	PTA	DC	None
6	64/M	Spoke injury	Left heel	Vastus lateralis muscle-chimeric double skin paddle	Flap 1: 9 × 5.5; Flap 2: 9.5 × 4.5; Muscle flap: 6 × 3 × 1	PTA	DC	None
7	44/M	Post-traumatic ulcer	Left heel	Vastus lateralis muscle-chimeric double skin paddle	Flap 1: 12 × 9; Flap 2: 16.5 × 8; Muscle flap: 10 × 3 × 2	ATA	DC	None
8	19/M	Traffic accident	Right heel	Microdissected thin vastus lateralis muscle-chimeric double skin paddle	Fap 1: 15 × 7; Flap 2: 9 × 6; Muscle flap: 8 × 5 × 1	PTA	DC	None
9	16/M	Traffic accident	Left heel	Microdissected thin double skin paddle	Flap 1: 18 × 7; Flap 2: 13.5 × 7.5	PTA	DC	None
10	21/M	Traffic accident	Right heel	Double skin paddle	Flap 1: 18 × 8; Flap 2: 13 × 8	PTA	DC	None
11	30/F	Traffic accident	Right heel	Double skin paddle	Flap 1: 16 × 8.5; Flap 2: 12 × 7	PTA	DC	None
12	52/M	Traffic accident	Left heel	Vastus lateralis muscle-chimeric double skin paddle	Flap 1: 14 × 8; Flap 2: 14 × 7; Muscle flap: 8 × 3 × 1	PTA	DC	None
13	20/M	Traffic accident	Left heel	Double skin paddle	Flap 1: 20 × 8; Flap 2: 14 × 6	PTA	DC	None
14	35/M	Traffic accident	Right heel	Double skin paddle	Flap 1: 16 × 7; Flap 2: 10 × 6	PTA	DC	None
15	35/M	Traffic accident	Right heel	Microdissected thin double skin paddle	Flap 1: 15 × 8; Flap 2: 14 × 7	ATA	DC	None
16	61/F	Post-traumatic ulcer	Left heel	Flow-through Microdissected thin double skin paddle	Flap 1: 13 × 7; Flap 2: 12 × 7	Flow through bridge PTA	DC	None

*F, female; M, male; ATA, anterior tibial artery; PTA, posterior tibial artery; DC, direct close*.

### Flap Design

**Figure 1:**
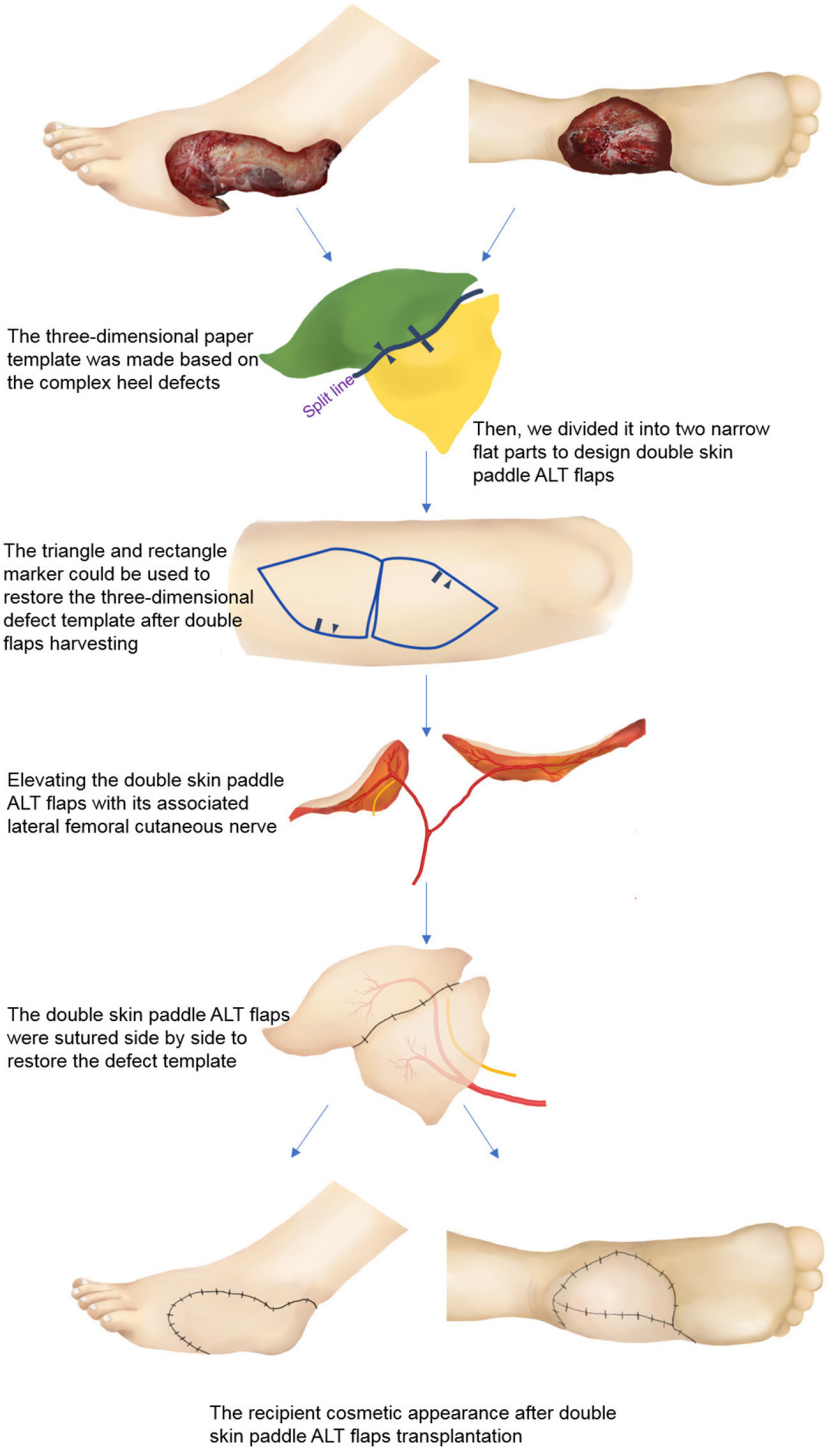
Schematic diagram of double skin paddle ALT flaps for complex heel defects. ALT, anterolateral thigh perforator.

Lower extremity computed tomography angiography (CTA) was carried out on all the patients to evaluate the location of perforators and vascular anatomy of the recipient site (13, 14). A handheld Doppler was used to verify the location of the perforators and guide the design of the free double skin paddle ALT flaps. Following radical debridement, a three-dimensional paper template was created replicating the shape of heel defect (Figure 1). The template was then divided into two narrow flat parts. Variants of the double paddle ALT flaps were designed based on the depth of the wounds. If a wound is superficial, a double skin paddle ALT flap can be chosen to repair the defect and achieve a good aesthetic outcome. For wounds with dead space, vastus lateralis (VL) muscle-chimeric double skin paddle ALT flaps can be used to repair superficial wounds and fill the dead space simultaneously. The microdissected thin technique was employed to improve the appearance if the flap appeared bulky. The flow-through method was used to bridge defective arteries where needed.

### Surgical Technique

Using a technique previously reported, double skin paddle ALT flaps were harvested (15). In brief, the retrograde tracing method was used to elevate the flaps, which were harvested in the superficial fascia layer. Two or more perforators were kept during flap harvest until the targeted perforator was identified for each skin paddle. The deep fascia was opened, and targeted perforator vessels following their intramuscular course were dissected aided by a surgical loupe. If the flaps were used to cover the weight-bearing area, the lateral femoral cutaneous nerve (LFCN) was preserved during the harvest. The perforators were traced back to the main trunk of the descending branch of lateral circumflex femoral vessels (LCFVs). If a dead space was present, to fill it, the distal end of LCFVs was used to harvest the muscle flap.

Following successful flap harvesting, the donor site was closed directly after achieving complete hemostasis, and a vacuum drain was placed. The flaps were transferred to the recipient site. Sensory nerve coaptation was performed in the reconstruction of weight-bearing regions. The descending branch of LCFVs was anastomosed to the vessels of a recipient with the end-to-side or end-to-end anastomosis technique.

Postoperatively, the extremities were kept warm and elevated with a temporary kickstand external fixator (16, 17). The flaps were monitored with hourly checks including evaluation of color, capillary refill, turgor, and surface temperature. The patients also received prophylaxis against deep vein thrombosis, multimodal pain management, and appropriate antibiotics. Static two-point discrimination (s2PD) test and Semmes–Weinstein monofilament (SWM) test were performed during follow-up visits to evaluate the sensory recovery of the flaps. A visual analog scale (VAS) score was used by the patients themselves to assess the cosmetic appearance of the heel after reconstruction. A score of 0 was rated as poor cosmetic outcome, and 10 was rated as excellent cosmetic outcome.

## Results

A total of 16 patients who had reconstructive heel surgeries were included in the study. Eleven patients underwent reconstruction with double skin paddle ALT flaps, and the rest received VL muscle-chimeric double skin paddle ALT flaps. While the microdissected thin technique was used in four patients to remove excess fat tissue that might lead to a swollen appearance, the flow-through technique was employed in two patients to bridge the posterior tibial artery. The size of the skin flaps ranged from 9.5 × 4.5 cm^2^ to 22 × 10 cm^2^, and the size of the muscle segments ranged from 6 × 3 × 1 cm^3^ to 10 × 3 × 2 cm^3^. No flap necrosis was observed. All donor sites were closed primarily after the harvest. The mean follow-up time was 22.6 months (range: 10–81 months). The wounds healed well, and the flaps provided reliable soft tissue coverage and good contour in the reconstructed areas. All the patients were able to ambulate independently during follow-up visits. At the last follow-up, the average s2PD of the flap was 32.7 mm (range: 27–37 mm). SWM test showed that 14 of the patients regained protective neural sensation. The mean VAS score was 8.8 (range: 8–9.5) (Table 2).

**Table 2:** Follow-up aesthetic and sensory outcome evaluation.

**Case**	**Follow-up (months)**	**s2PD (mm)**	**5.07 SWM (10-Gram)**	**VAS Score (Appearance)**
1	18	30	Positive	9
2	12	27	Positive	9
3	18	32	Positive	8.5
4	28	35	Positive	9.5
5	12	29	Positive	8
6	12	33	Negative	9
7	10	32	Positive	8
8	15	36	Positive	9
9	54	34	Positive	9.5
10	12	32	Positive	8.5
11	10	30	Positive	8
12	13	36	Positive	8.5
13	30	33	Positive	9
14	16	35	Negative	8
15	20	37	Positive	9.5
16	81	33	Positive	9.5

*s2PD, static two-point discrimination test*.

*SWM, Semmes–Weinstein monofilament test*.

*VAS, visual analog scale*.

### Case Reports

#### Case 2

**Figure 2:**
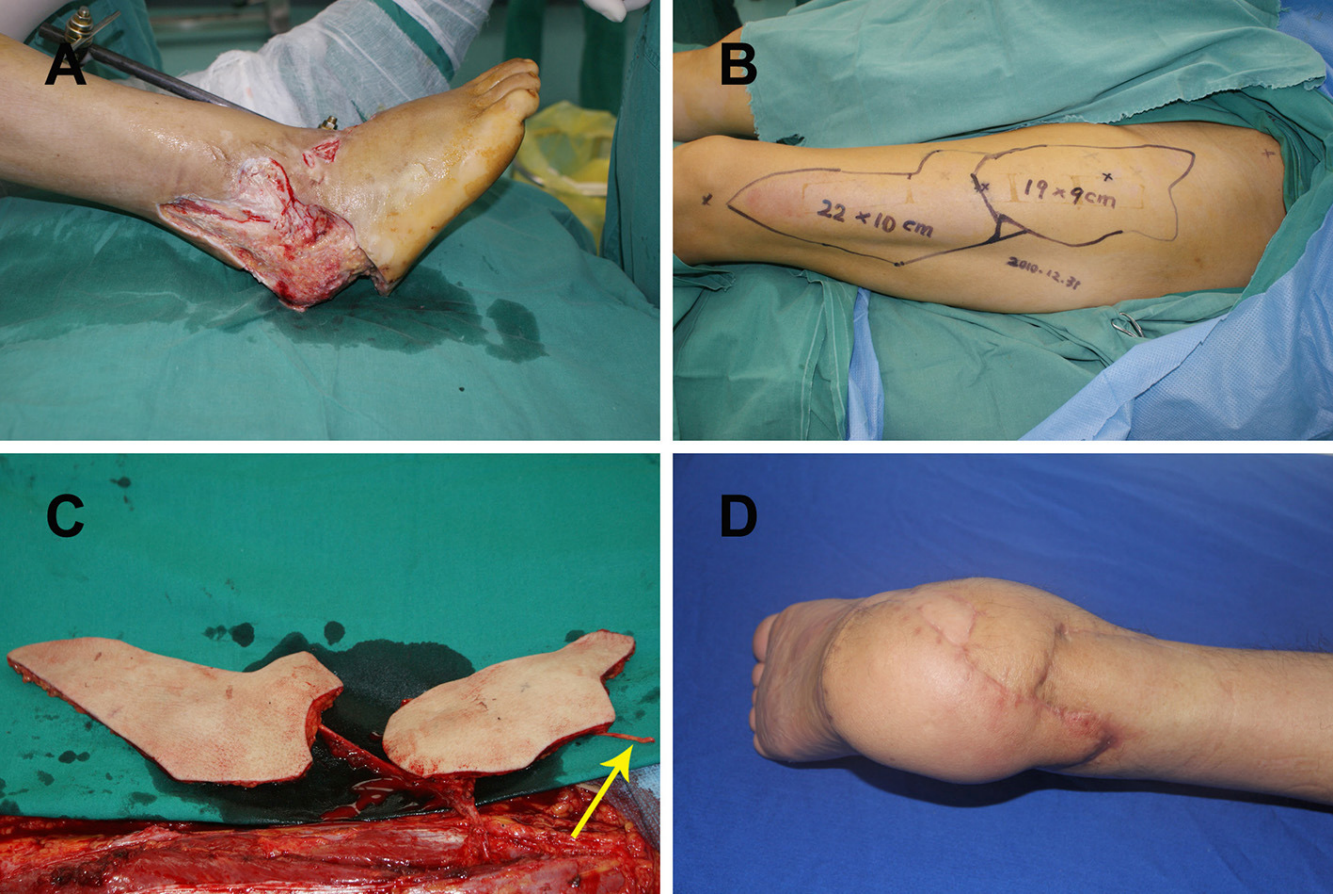
**(A)** Large right heel defect following radical debridement; **(B)** double skin paddle ALT flap design; **(C)** elevating the double skin paddle ALT flaps showing the lateral femoral cutaneous nerve (yellow arrow); **(D)** postoperative view of the recipient site after 12 months. ALT, anterolateral thigh perforator.

A 25-year-old man presented with a large soft tissue defect of the right heel after a motor vehicle injury. Radical debridement was performed, which left a large soft-tissue defect (Figure 2A). A defect template was created and divided into two narrow flat parts. A double skin paddle ALT flap was designed based on the defect template. The size of flaps 1 and 2 was 22 × 10 cm^2^ and 19 × 9 cm^2^, respectively (Figure 2B). During harvest of the flaps, the LFCN was dissected as the weight-bearing area was being repaired (Figure 2C). LCFVs were anastomosed to the posterior tibial artery and accompanying vein at the recipient site. End-to-end epineurial repair was conducted between the ALT cutaneous nerve and the medial plantar cutaneous nerve. The postoperative course of the patient was uneventful, and the recipient site had a satisfactory contour at 12-month follow-up (Figure 2D).

#### Case 8

**Figure 3:**
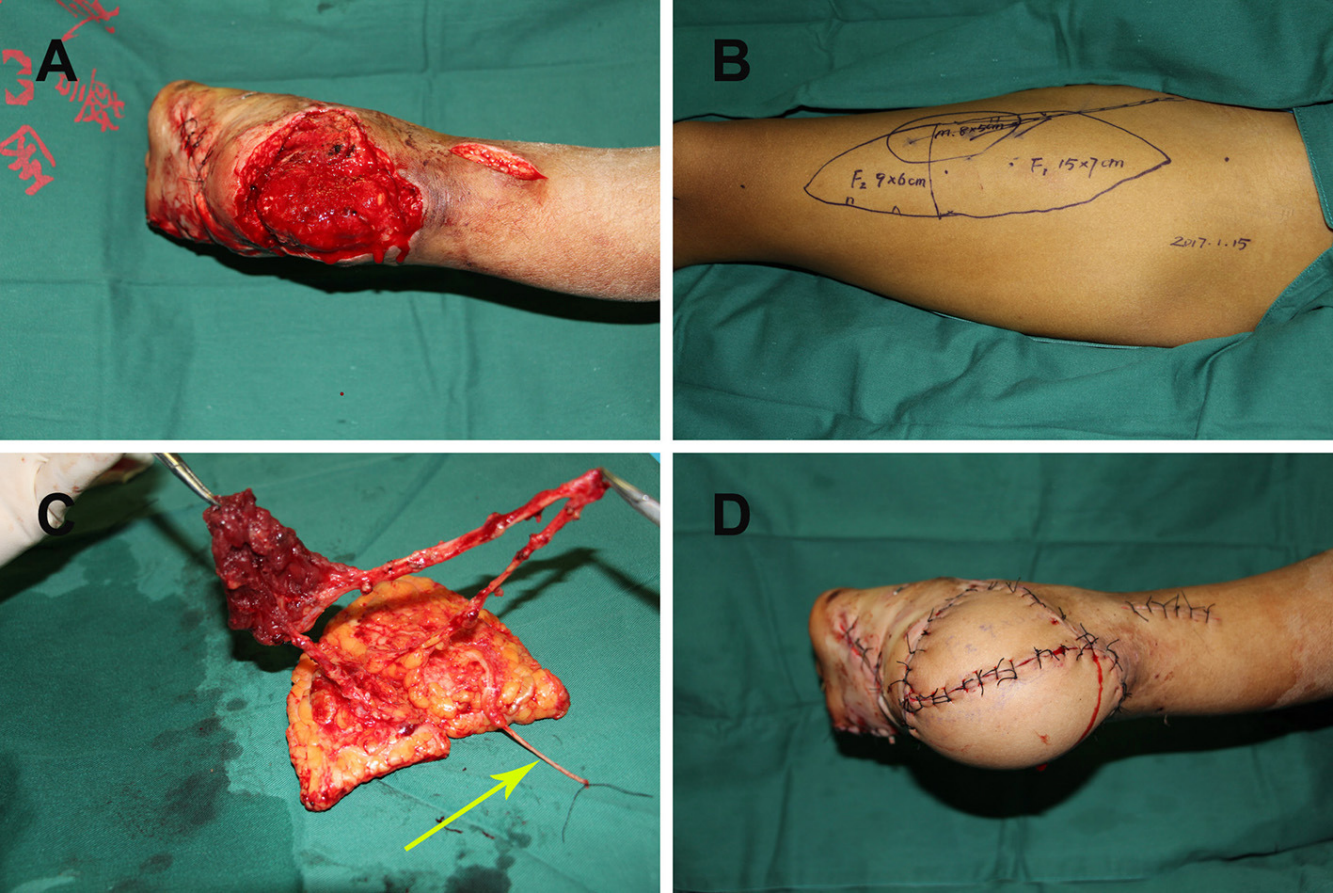
**(A)** Right heel soft tissue defect with dead space following radical debridement; **(B)** chimeric double skin paddle ALT flap design; **(C)** harvesting of chimeric double skin paddle ALT flaps showing the lateral femoral cutaneous nerve (yellow arrow); **(D)** postoperative view of the recipient site. ALT, anterolateral thigh perforator.

A 19-year-old man suffered a traffic injury and presented with a right heel soft-tissue defect with dead space (Figure 3A). A chimeric double skin paddle ALT flap was designed (Figure 3B). The size of flap 1 was 15 × 7 cm^2^, the size of flap 2 was 9 × 6 cm^2^, and the muscle paddle measured 8 × 5 × 1 cm^3^ (Figure 3C). The flaps were placed side by side to cover the heel defect, and the muscle flap filled the dead space. Flap LCFVs were anastomosed to the posterior tibial artery and accompanying vein at the recipient site. End-to-end epineurial repair was conducted between the ALT cutaneous nerve and the medial calcaneal nerve. The postoperative course of the patient was uneventful, and the contour of the recipient site was satisfactory (Figure 3D).

## Discussion

Large heel defects with or without significant dead space are still constituting a challenge for microsurgeons. Thus, there is a need to employ new reconstructive techniques (18, 19). An ideal reconstruction should cover large defects, completely fill the dead space, restore protective sensation to prevent ulcer formation, and achieve a good cosmetic appearance. The special structure of soles makes local flaps a viable option in restoring heel function and aesthetic appearance based on the “like with like” plastic surgery principle (20–22). However, local flaps are limited by complex defects, such as large wounds with or without dead space (23, 24). On the other hand, free flaps are a feasible option for reconstruction of large heel defects (3). Therefore, to bypass challenges associated with the use of local flaps for reconstruction of complex defects and to be able to restore heel shape, we explored the use of free double skin paddle ALT flaps.

The most frequently used free flaps are radial forearm flaps, ALT, and thoracodorsal artery perforator flaps (25–28). Jachna et al. reported using radial forearm free flaps to cover lateral heel wounds (29). They showed that all flaps survived with good functional and cosmetic results and concluded that radial forearm flaps are a reliable option to repair heel wounds. However, poor appearance of the donor site and need to sacrifice the radial artery limit its application. In contrast, free ALT flaps and thoracodorsal artery perforator flaps used to repair large heel defects have the advantage of low donor site morbidity (30). However, they are not very effective in restoring the complex shape of heels. Pan et al. used a waveform design to repair a heel with a good shape and cosmetic appearance (31). This design adopted the flap economy concept with minimal damages to the donor site. However, it does not apply to all situations (32). From our experience, large heel defects can be divided into two narrow flat defects organized in three dimensions to achieve a better contour of heels. This study demonstrated good aesthetic appearance and restoration of sensation in reconstruction of large heel defects using double skin paddle ALT flaps. All the patients had viable non-bulky flaps, were able to walk, and did not develop ulcers during the follow-up period.

The use of double skin paddle ALT flaps for large heel defects has the following advantages. First, double skin paddle ALT flaps enable restoration of the complex shape of heels. Second, double skin paddle ALT flaps are versatile for large defects, VL muscle-chimeric flaps can be used to fill dead spaces, the flow-through technique can bridge the defective artery, and the microdissected thin technique can remove excess fat tissues to improve the cosmetic appearance. The versatility of free double skin paddle ALT flaps for complex heel defects achieves good outcomes with less donor site morbidity. Third, restoration of heel sensation and ability to resist pressure can be achieved by carrying out end-to-end neurorrhaphy.

Nevertheless, these flaps have limitations, and they should be used with caution. It should be taken into account that perforator variability may influence flap design (33, 34). For our patients, CTA scan was performed before the surgery, and three-dimensional CTA reconstruction was performed to locate and identify perforators. A handheld Doppler was also used to verify the location of perforators, which significantly reduces the incidence of flap necrosis. However, if double skin paddle ALT flaps cannot be harvested intraoperatively, sequential flaps can be used instead. The defect template also needs to be carefully evaluated. Surgeons should have a three-dimensional understanding of flap design and be familiar with intra-muscular dissection. Rectangular and triangular markings were used in this study to ensure that the tailored double-skin flap will restore the original heel shape (Figure 1). The harvest and insertion of two skin paddles may increase the risk of kinking or twisting of the vascular pedicle; thus, extra caution should be taken. Besides, a temporary kickstand external fixator should be applied to avoid pressure on the flaps. Lastly, both microdissected thin technique and suprafascial elevation can be used to harvest thin flaps, which can be chosen based on the experience and preference of a surgeon.

The primary limitation of this study is the small number of cases. There was no comparison group as well. Therefore, further studies comparing double skin paddle ALT flaps and other flap techniques should be carried out.

## Conclusion

Double-skin paddle ALT flaps are versatile for reconstruction of complex heel defects. In addition to providing coverage and pleasing contour, regain of protective sensation makes it a favorable option for heel reconstruction.

## Data Availability Statement

The raw data supporting the conclusions of this article will be made available by the authors, without undue reservation.

## Ethics Statement

The studies involving human participants were reviewed and approved by Xiangya Hospital. The patients/participants provided their written informed consent to participate in this study.

## Author Contributions

Study conceptualization was performed by JH and JT. Data collection was performed by JH, GG, PW, FY, and LQ. The first draft was written by JH. Data analysis and review and editing were performed by all the authors.

## Conflict of Interest

The authors declare that the research was conducted in the absence of any commercial or financial relationships that could be construed as a potential conflict of interest.

## Publisher's Note

All claims expressed in this article are solely those of the authors and do not necessarily represent those of their affiliated organizations, or those of the publisher, the editors and the reviewers. Any product that may be evaluated in this article, or claim that may be made by its manufacturer, is not guaranteed or endorsed by the publisher.
